# Empirical rovibronic energy levels of C_3_

**DOI:** 10.1080/00268976.2023.2276912

**Published:** 2023-11-02

**Authors:** Jonathan Tennyson

**Affiliations:** Department of Physics and Astronomy, University College London, London, UK

**Keywords:** Rovibronic energy levels, carbon trimer, MARVEL

## Abstract

The carbon trimer, ${\text{C}}_{3}$ also known as propadienediylidene, is a quasi-linear molecule with an unusual electronic structure and a very flat bending potential in its ground electronic state. ${\text{C}}_{3}$ is an important species in astrophysics and carbon plasmas. Observed transition wavenumbers within and between the $\tilde{X}{\;}^{1}\Sigma_{g}^{+}$ and $\tilde{A}{\;}^{1}\Pi_{u}$ states of ${\text{C}}_{3}$ are extracted from 21 papers and then subjected to a Measured Active Rotational-Vibrational Energy Levels (MARVEL) analysis: a corrected list of 4940 transitions are inverted to yield 1887 empirical energy levels. Uncertainties for these levels are determined using a newly implemented bootstrap method. These levels will provide input for developing a full spectroscopic model for ${\text{C}}_{3}$ which can used to generate a line list for the $\tilde{X}{\;}^{1}\Sigma_{g}^{+}$ and $\tilde{A}{\;}^{1}\Pi_{u}$ states.

## GRAPHICAL ABSTRACT

**Figure:**
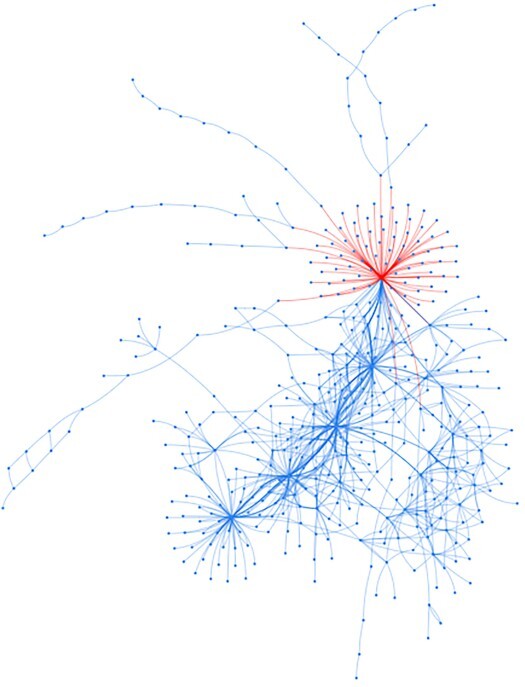

## 1. Introduction

The carbon trimer, ${\text{C}}_{3}$, which is also known as propadienediylidene is a quasi-linear molecule with an unusual electronic structure and a very flat bending potential. In common with the carbon dimer, C ${}_{2}$, ${\text{C}}_{3}$ is often described as a radical, e.g. [1, 2], but actually it has a closed shell ${}^{1}\Sigma_{g}^{+}$ electronic ground state. ${\text{C}}_{3}$ is formed in carbon plasmas where its spectra has been recorded in vapour deposition of fluorocarbon and hydrocarbon films [2], laser-generated graphite plasma [3] and diamond deposition [4]. Similarly, ${\text{C}}_{3}$ is prominent in space where its spectrum has been detected in comets [5], the interstellar medium [6–10], the circumstellar shell of IRC + 10216 [11], in external galaxies [12], and, notably, in the atmospheres of cool carbon stars [13, 14].

There physical importance and unusual properties of ${\text{C}}_{3}$ has stimulated have a large number high resolutions spectroscopic studies. Here we are interested in studies involving the $\tilde{X}{\;}^{1}\Sigma_{g}^{+}$ ground state and $\tilde{A}{\;}^{1}\Pi_{u}$ first excited singlet state of the molecule. The previous studies are therefore divided into three categories. Those used in the measured active rotational-vibrational energy levels (Marvel) study presented below [15–35], those which did not provide any new data on measured transitions [7, 36–45] and studies which consider higher singlet and triplet electronic states of the molecule [46–50].

Marvel provides a means of representing spectroscopic data via energy levels which is essentially model free; that is the method makes no assumptions beyond the simple energy difference rules linking different energy levels in a bound-state quantum mechanical system. Thus, for example, the effects of anharmonicity or accidental resonances between states which are commonly observed in molecular spectra do not pose any particular problem with the Marvel formalism. During the course of this work Martin-Drumel *et al.* [35] provide a comprehensive effective Hamiltonian analysis of ${\text{C}}_{3}$, which provides a complimentary study to the work presented here; as is often the case with such studies they omitted or artificially increased the uncertainties of some measured transitions when performing their fits to allow them to cope with the effects of perturbations. Within Marvel such data are only omitted if they are found to be inaccurate or incorrect. The Marvel methodology of Császár and co-workers [51] is outlined and discussed in the following section. Section 3 considers the application to the ${\text{C}}_{3}$ molecule with results presented and discussed in Section 4. Section 5 presents conclusions.

## 2. Marvel

The Marvel algorithm used in this study is based on the theory of spectroscopic networks [52–55] where the energy levels form the nodes and linking edges are the transition data. By collecting all measured transitions for a given isotopologue, the Marvel procedure can be used to perform an inversion to provide energy levels. This sounds simple but there are a number of practical problems.

To be used in a Marvel study, it is necessary for each transition to be both assigned and to have an associated uncertainties. The assignments need to use a consistent set of quantum number which can involve re-assigning or re-analysing the given quantum numbers, see Ref. [56] for example. Uncertainty handling is often itself an issue. Absolute uncertainties often vary by several orders of magnitude within a given Marvel dataset. Furthermore, stated uncertainties are often found to be optimistic or only given for the best lines and spectra, particularly older ones, sometimes need recalibration. Use of these uncertainties in the Marvel procedure has proved to be quite challenging with the changes between the various versions of Marvel [56–59] being largely driven by the desire to improve the treatment of uncertainties. The most recent implementation, which is employed here, is MARVEL4 (T. Furtenbacher, private communication, 2023) which uses a bootstrap procedure to determine realistic uncertainties for the empirically-determined energy levels [56].

The Marvel methodology was originally developed [57] to aid the systematic analysis and synthesis of spectra of various water isotopologues as part of a IUPAC task group [60–64]. One result of this work was the realisation that use the empirical energy levels determined by the Marvel led to a large increase in the number of transitions wavenumbers that could be predicted to spectroscopic accuracy; this property has proved to be a useful feature of other Marvel studies [65–67]. Marvel energy levels can also be used to provide partition functions and thermodynamic dats for the molecule concerned [68–70] as well as providing the data needed to fit accurate potential energy functions. It anticipated that the ${\text{C}}_{3}$ levels generate here will be used for all these purposes in due course.

Graph theory can be used to help identify specific experimental measurements which would improve the accuracy and robustness of the spectroscopic network (SN) for a given molecule [71]. This idea is being used in modern, ultra-high resolution studies [72–74] as a means to improve the results of Marvel study.

## Application to ${\text{C}}_{3}$

3.

### 3.1. Quantum numbers

The electronic structure of the excited states of ${\text{C}}_{3}$ is quite complicated with Renner-Teller, Jahn-Teller and pseudo-Jahn-Teller interactions all identified in the low-lying excited electronic states [75]. However, only for the ground $\tilde{X}{\;}^{1}\Sigma_{g}^{+}$ state and first excited $\tilde{A}{\;}^{1}\Pi_{u}$ state are there sufficient spectroscopic data to justify a Marvel study. We therefore concentrate on these states here.

For the $\tilde{X}{\;}^{1}\Sigma_{g}^{+}$ state the conventional (linear-molecule) harmonic oscillator quantum numbers are $\nu_{1}$: symmetric stretch with fundamental frequency of 1223.6 cm ${}^{-1}$ [37], $\nu_{2}$: degenerate bending mode at 63.4 cm ${}^{-1}$ [35] and $\nu_{3}$: asymmetric stretch at 2040.0 cm ${}^{-1}$ [17]. The very low bending excitation due to the very flat potential about the (quasi-)linear equilibrium geometry of ${\text{C}}_{3}$ has a number of consequences. It means that detection of interstellar ${\text{C}}_{3}$ unusually used the bending excitation which lies in the terahertz region [7]. Even though the bending mode shows an unusual anharmonicity which leads to spacing between states growing with bending excitation [35], another consequence is that the stretching states overlap many highly excited bending states with the increased possibility of perturbations due to accidental resonances.

The degeneracy of the $\nu_{2}$ bending mode means one has to also consider a vibrational angular momentum, $\ell_{2}$. $\ell_{2}$ takes positive values with $\ell_{2}=v_{2},v_{2}-2,v_{2}-4,\ldots$. For the $\tilde{X}{\;}^{1}\Sigma_{g}^{+}$ state we set quantum number $\ell =\ell_{2}$. Therefore, the vibrational states can be labelled $\left(v_{1}v_{2}^{\ell }v_{3}\right)$ with the ground state given by $\left(00^{0}0\right)$. There is an alternative and probably more widely used notation which labels states with ℓ=0,1,2,3,… using the Greek letters Σ,Π,Δ,Φ,…. In some works the Greek letter designations are augmented g or u subscripts to denote gerade or ungerade but these add no new information as they simply denote whether $v_{3}$ is even or odd, respectively. Here the $\left(v_{1}v_{2}^{\ell }v_{3}\right)$ notation is adopted. Levels in the $\tilde{X}{\;}^{1}\Sigma_{g}^{+}$ state with ℓ=0 all have e rotationless parity; while for ℓ>0 both e and f states are possible.

The last quantum number is the standard angular momentum, *J*, which obeys the rule J≥ℓ. However, for ${}^{12}{\text{C}}_{3}$ an additional consideration arises. ${}^{12}$C is a Boson with a nuclear spin of zero. As a consequence of the Pauli Principle, states of e parity in the $\tilde{X}{\;}^{1}\Sigma_{g}^{+}$ state only exist when with $J+v_{3}+\ell$ is even; the f states occur for cases where ℓ>0 and $J+v_{3}+\ell$ is odd.

The $\tilde{A}{\;}^{1}\Pi_{u}$ state is a Renner-Teller system which splits for bent geometries. However, as for the ground state, linear geometry quantum numbers have been shown to provide a practical method of representing the levels in $\tilde{A}{\;}^{1}\Pi_{u}$ state. Note Marvel is agnostic on this topic; the only requirement of Marvel is that is level has a unique set of quantum numbers. The same quantum numbers are therefore adopted for the $\tilde{A}{\;}^{1}\Pi_{u}$ state as the $\tilde{X}{\;}^{1}\Sigma_{g}^{+}$ with a simple extension to allow the fact at Λ≠0. For the $\tilde{A}{\;}^{1}\Pi_{u}$ state $v_{1},v_{2},\ell_{2},v_{3},J$ and e/f all have the same definition as for the $\tilde{X}{\;}^{1}\Sigma_{g}^{+}$ state, but the definition of ℓ needs to be generalised to allow for the electronic angular momentum represented by |Λ=1| for a Π electronic state. In this case, when $\ell_{2}>0$, $\ell =\ell_{2}-1$ or $\ell_{2}+1$, denoted respectively m and p below; while if $\ell_{2}=0$, ℓ=1. However, if $v_{2}\geq 3$ then it is possible to obtain two ℓ=0 states starting from $\ell_{2}=1$; these states are conventionally denoted $\Sigma^{+}$ and $\Sigma^{-}$; here they are called 0p and 0m, respectively. Similarly when $0<\ell <\ell_{2}$ there are also two ways of reaching each ℓ values so states ± labels are also needed. For example, if $v_{2}=2$ then a state with ℓ=1 can be reached either from $\ell_{2}=0$ or 2, such states conventionally labelled $\Pi^{+}$ or $\Pi^{-}$, or here 1p and 1m. For the $\tilde{A}{\;}^{1}\Pi_{u}$ state allowed *J* states follow the same rule as for the $\tilde{X}{\;}^{1}\Sigma_{g}^{+}$ state, namely levels $J+v_{3}+\ell$ even are e and those $J+v_{3}+\ell$ are odd, since the extra parity due to Λ is absorbed into ℓ. This means that means for each state ℓ>0 there is only either an e or an f level for each *J*.

Besides the $\tilde{X}{\;}^{1}\Sigma_{g}^{+}$ and $\tilde{A}{\;}^{1}\Pi_{u}$ states, there are some observed perturbations due other electronic states which lead to extra observed transitions. Some of these perturbations can be associated with the $\tilde{a}{\;}^{3}\Sigma_{u}^{-}$ state but most have not been assigned to a definitive electronic state; these are labelled P=1, P=1e and P=1f following the cited studies. While the *J* and parity values of the perturbing state are known because they have to be same as those of the state being perturbed, other quantum numbers such as vibrational state or fine structure component are not known for any of the perturbations. It was therefore decided to keep all quantum numbers except the state quantum number the same for both interacting states; this convention makes interacting energy levels easy to identify.

Finally, ${\text{C}}_{3}$ obeys standard dipole selection rules: ΔJ=0 and e ↔ f, not J=0↔0; or ΔJ=±1 and e ↔ e or f ↔ f. Transitions from the ground state to the symmetric stretching fundamental (and overtones) are forbidden making them hard to study.

### 3.2. Sources

We extracted data from 21 sources [15–35], see Table 1. Notes on these sources are given below.

**Table 1.** Experimental sources used to construct the ${}^{12}{\text{C}}_{3}$ Marvel spectroscopic network. A and V give the number of actual and validated transitions considered for each source; uncertainty statistics (in cm ${}^{-1}$) are AIU = average initial uncertainty, AMR = average MARVEL reproduction of the source's lines, and MR = maximum reproduction in the source.

Tag/reference	Range (cm ${}^{-1}$)	A/V	AIU	AMR	MR
03GePeGiWi [25]	63.062–66.874	10/10	1.684e−05	3.618e−06	0.215
03McCaAsSa [26]	24671.060–24683.200	24/24	6.042e−03	8.989e−03	1.488
05TaHiAmBe [27]	24560.866–24685.786	71/65	7.077e−03	4.363e−03	0.617
05ZhChMeHs [28]	23440.310–24684.656	85/84	1.286e−02	3.563e−03	0.277
11ChZhMeHs [29]	23378.320–23395.234	29/29	1.103e−02	2.301e−03	0.209
13KrLuEnKe [30]	3233.819–3280.603	57/57	9.737e−04	3.438e−04	0.353
14HaZhLiUb [31]	24651.730–24685.270	58/57	2.000e−02	1.549e−02	0.774
14ScKrGaZh [32]	24659.999–26351.854	78/71	1.042e−02	1.170e−02	1.123
16BrBuScLu [33]	51.357–80.303	17/17	1.412e−04	4.822e−05	0.341
18ScDoSeZh [34]	3109.856–3274.077	281/185	2.000e−03	6.479e−04	0.324
23MaQiDoPi [35]	1919.934–26353.232	2097/2042	3.715e−03	1.809e−03	0.487
23MaQiDoPi_EH	377.523–1561.878	8/8	5.000e−03	0.000e+00	0.000
65GaHeLaRo [15]	24298.460–25770.140	1082/992	3.461e−02	2.816e−02	0.814
67Merer [16]	23314.640–245.720	70/69	6.957e−02	8.335e−02	1.198
88MaKaKaHi [17]	2024.425–2087.845	28/26	2.000e−03	7.238e−04	0.362
89KaMaKaHi [18]	1975.426–2129.641	173/173	1.000e−03	4.919e−04	0.492
90RoGo [19]	4038.200–4571.200	70/70	5.000e−01	0.000e+00	0.000
90ScCoPuHe [20]	58.078–69.502	7/7	1.157e−04	2.919e−05	0.252
94BaCaPrQi [21]	25589.870–26852.710	159/94	1.000e−01	1.326e−01	1.326
95IzYa [22]	23149.743–24028.437	83/83	2.795e−02	1.986e−02	0.710
97ToCh [23]	24869.899–25131.582	320/290	1.274e−02	4.764e−03	0.374
98IzYa [24]	24231.357–29671.215	133/133	7.669e−02	7.388e−02	0.963

The recent study by Martin-Drumel *et al.* (23MaQiDoPi) [35] also conducted a comprehensive review of many, but not all, of the sources considered here. They recalibrated and/or recommended different uncertainties for many of the sources they considered. Generally these recommendations were adopted here. They also excluded a significant number of transitions from their effective Hamiltonian fit. These transitions were included in the Marvel analysis and generally validated successfully. This difference is probably a reflection of perturbations which can be difficult to model in effective Hamiltonian fits but do not cause problems with Marvel. Other source specific comments are:

65GaHeLaRo [15]: Data taken from 23MaQiDoPi who did some recalibration, (re-)set uncertainties and made a few re-assignment.

67Merer [35]: Taken from 23MaQiDoPi who made a partial recalibration.

88MaKaKaHi [17]: No uncertainty was given in the paper; 0.001 cm ${}^{-1}$ was originally adopted from 23MaQiDoPi but this value gave a lot of bad lines so 0.002 cm ${}^{-1}$ was used. 23MaQiDoPi omit three lines; the original Marvel runs omitted three different lines; in the end only two lines were not validated.

89KaMaKaHi [18]: No uncertainty was given in the paper; 0.001 cm ${}^{-1}$ was adopted from 23MaQiDoPi. 23MaQiDoPi omit 24 lines (given in brackets as calculated omitted); however, once line 89KaMaKaHi.91 was corrected to 1993.6655 cm ${}^{-1}$ following 23MaQiDoPi, all lines were validated.

90ScCoPuHe [20]: 23MaQiDoPi doubled the uncertainty of the of P(2) line; this was not found to be necessary.

90RoGo [19]: performed stimulated-emission pumping spectroscopy which probed excited states within the ${\tilde{X}}^{1}\Sigma_{g}^{+}$ state by initially pumping strong transitions to ${\tilde{A}}^{1}\Pi_{u}$ state and the dumping them back to ground state. This technique can access vibrationally excited states which are hard to access using single photon techniques. Unfortunately the transition data for this study are no longer available; instead the 70 ${\tilde{X}}^{1}\Sigma_{g}^{+}$ state energy levels with J≤12 presented in the table were included as (pseudo-)transitions from the ${\tilde{X}}^{1}\Sigma_{g}^{+}$ $\left(00^{0}0\right)$ level. These are relatively low accuracy measurements and the levels/transitions were given an uncertainty of 0.5 cm ${}^{-1}$.

94BaCaPrQi [21]: 23MaQiDoPi suggests that 94BaCaPrQi's transitions are misassigned by comparison with 67Merer and 87ToCh. MARVEL analysis agrees that the (02 ${}^{\ell }$0)-(000) and (00 ${}^{0}$2)-(000) bands do not validate and all lines in these bands were removed. The other bands were retained as they appear to be self-consistent, that is the assignments obey combination differences. Some further experimental work on this bands would be useful to confirm the reliability of these data.

97ToCh [23]: 23MaQiDoPi increases uncertainties for perturbed lines by factor of 10. This increase seems to be necessary to accommodate the effect of perturbations in the fit rather than any measurement uncertainty so the original uncertainties were used unchanged.

95IzYa [22] and 98IzYa [24]: data for sources 95IzYa and 98IzYa are all given in 98IzYa. No uncertainties are given in either paper: initial ones were taken from 23MaQiDoPi but some had to be increased.

03McCaAsSa [26]: the recalibration by 05TaHiAmBe was adopted by 23MaQiDoPi and here. Some extra assignments to perturbing states by 05TaHiAmBe and 05ZhChMeHs were adopted here.

05TaHiAmBe [27]: lines due to 03McCaAsSa lines were removed from this source. Line 05TaHiAmBe.6 was reassigned to:

A1Piu 0 0 1 0 32 f X1Sigmag+ 0 0 0 0 32 e

14ScKrGaZh [32]: vacuum wavenumbers and uncertainties were taken from 23MaQiDoPi. Perturbed lines are assigned to $\tilde{a}{\;}^{3}\Sigma_{u}^{-}$ following 05ZhChMeHs. Line 14ScKrGaZh.1 was removed (labelled a misassignment by 23MaQiDoPi); six other lines were removed as they appear to be inaccurate.

18ScDoSeZh [34]: an uncertainty of 0.002 cm ${}^{-1}$ as in the paper was adopted. 23MaQiDoPi recommend 0.001 cm ${}^{-1}$ for most lines and 0.01 cm ${}^{-1}$ for others; it is unclear if this adjustment represents measurement uncertainty or reflects the quality of the effective Hamiltonian fit.

23MaQiDoPi [35]: the 10 transitions labelled as A000 – X020 transitions and assigned to $\tilde{a}{\;}^{3}\Sigma_{u}^{-}$ were re-assigned as P=1f.

 90NoSe [39]: this source was not included in the MARVEL compilation because, unfortunately, the transition data is lost (T Sears, private communication, 2022). However, Table 2 of the paper presents 41 terms values for levels with (0 16 ${}^{\ell }$ 0) with ℓ≤8 and J≤ 14. These levels can be used to augment the levels presented here.

**Table 2.** List of lowest-lying rotation-vibration states, all belonging to the $\tilde{X}{\;}^{1}\Sigma_{g}^{+}$ electronic state, in the main unconnected spectroscopic networks; *N* is the number of transitions in the network. The energies are those given by the effective Hamiltonian of 23MaQiDoPi [35], but no such energies are known for the states with $v_{1}\geq 1$.

$v_{1}$	$v_{2}$	ℓ	$v_{3}$	*J*	p	*N*	Level (cm ${}^{-1}$)
0	0	0	0	58	e	10	1561.878200
0	1	1	0	54	f	6	1367.458219
0	3	3	0	32	f	22	687.501410
0	4	2	0	36	e	9	904.941926
0	4	2	0	33	f	10	812.742469
0	4	4	0	41	f	5	1077.937168
0	5	1	0	40	f	5	1159.845882
0	5	5	0	5	e	64	377.523167
3	0	0	0	0	e	35	
4	0	0	0	0	e	21	
5	0	0	0	0	e	20	
6	0	0	0	0	e	9	
1	1	1	0	1	e	18	

## 4. Results

The original compilation of experimental data gave 4932 transitions which yielded 1754 energy levels from a main network of 4447 transitions. This meant 485 observed transitions were not part of the main network; inspection showed that there were a number of disconnected networks containing a significant number of transitions. Table 2 lists the lowest energy state and size of the larger disconnected networks; no other grouping had more than 3 unique transitions.

It was therefore decided to try to connect these major networks using artificial transitions generated from the effective Hamiltonian constants of 23MaQiDoPi [35] and programme PGOPHER [76]. Eight new (pseudo-)transitions, tagged 23MaQiDoPi_EH, were added with the wavenumbers given in Table 2, lower state $\tilde{X}{\;}^{1}\Sigma_{g}^{+}$ $\left(00^{0}0\right)$ state and an uncertainty of 0.005 cm ${}^{-1}$ were added. With these extra transitions the main spectroscopic network contained 4586 transitions and gave 1886 empirical energy levels. The MARVEL input (transitions) and output (energies) files are given in the supporting material. However, as implied by Table 2, transitions involving several $v_{1}$ overtone states remain unattached to the main network.

### 4.1. Empirical rovibrational energy levels

The energy of a total of 1887 levels were determined by Marvel; most of these levels are validated by combination differences but a total 241 levels were only linked to the main network by a single line. Such levels must be considered to be tentative.

**Figure 1.**
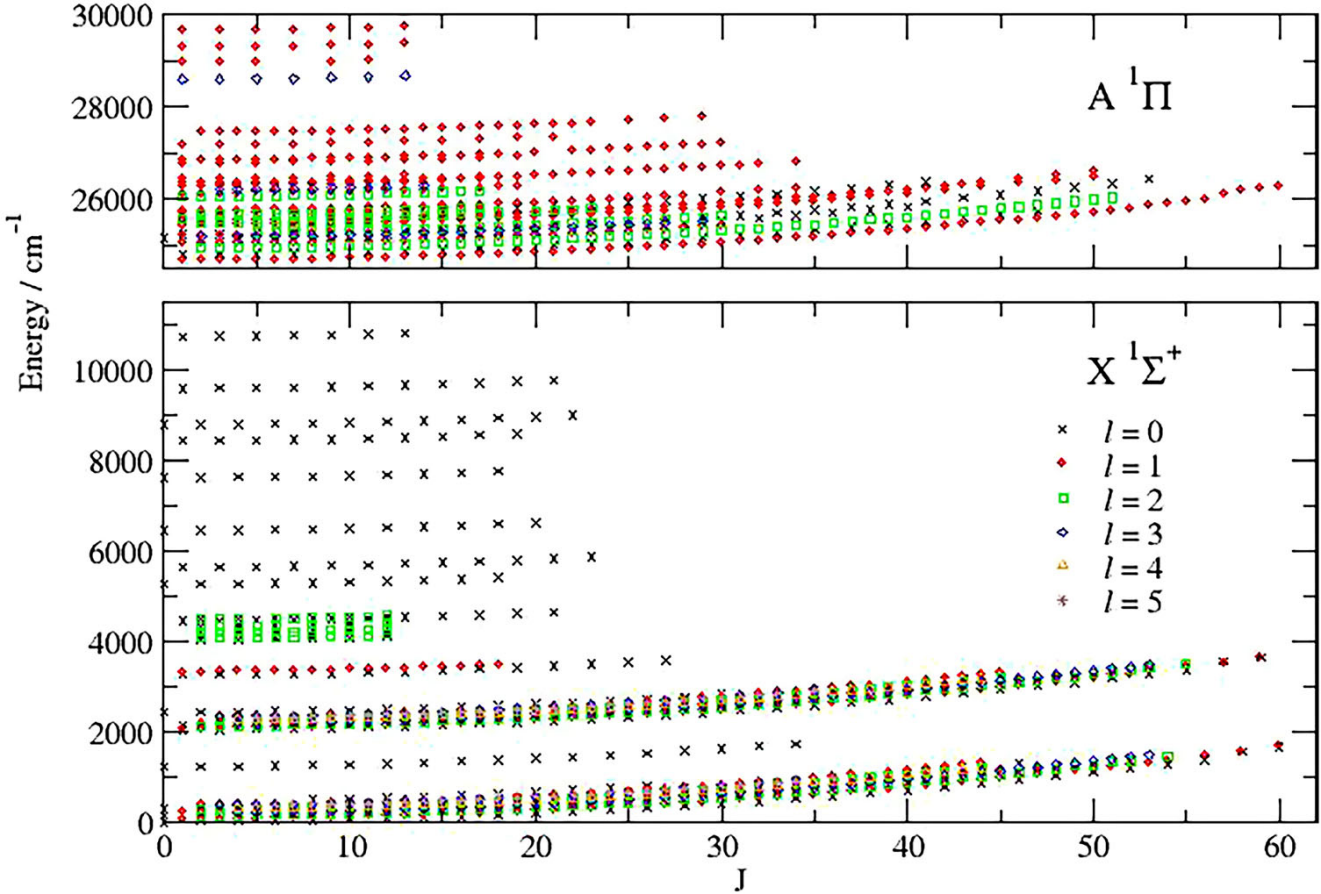
Overview of energy levels determined in this work designated by *J* and ℓ quantum numbers; the A ${}^{1}\Pi$ state levels include a number of extra levels due to interactions with other nearby states.

Figure 1 gives a graphical representation of the distribution of energy levels. It can be seen that while states up to ℓ=5 are characterised, the higher levels in the ground state all have ℓ=0 and correspond substantially to excited asymmetric ( $v_{3}$) stretching excitations. In fact Rohlfing [37] used laser-induced-fluorescence spectroscopy from the $\tilde{A}{\;}^{1}\Pi_{u}$ state to characterise a large number of highly excited bending states in the $\tilde{X}{\;}^{1}\Sigma_{g}^{+}$ state: up to $v_{2}=24$ at 2579.2±4.8 cm ${}^{-1}$ for $v_{1}=v_{3}=1$ and $v_{2}=37$ when $v_{3}=1$. However, the line data for these medium resolution spectra appears to be no longer available.

### 4.2. Uncertainties

Marvel has a variety of methods for characterising uncertainties; some these are given by source in Table 1. What this showed was that average uncertainty with which Marvel reproduces the lines from a given source (AMR) is generally in line with the average initial uncertainty (AIU); remembering that, as discussed in the notes on the sources, in several cases the AIU was increased from the value given by the original authors. However, maximum reproduction of the line by Marvel is rather large in some cases.

These measures of uncertainty discussed above all refer to the original lines. However, it is important to characterise the uncertainty in the energy levels produced by the Marvel procedure. Marvel4 introduced a bootstrap procedure for doing precisely this [56]. Running the bootstrap method gives uncertainties on average about 40% larger than those given by just running MARVEL, suggesting that some of the uncertainties used in the transition file remain optimistic. The bootstrap uncertainties are the ones given in the energy file provided in the supporting information. As can be seen from Table 3 discussed below, the uncertainties on different levels span several orders of magnitude.

**Table 3.** List of lowest energies for each vibrational state, $E(v_{1},v_{2}{,}^{\ell },v_{3}$; J=ℓ), in cm ${}^{-1}$, with uncertainties in parenthesis in units of the last digit; *n* is the number of transitions linking to the given state. For the $\tilde{X}{\;}^{1}\Sigma_{g}^{+}$ state, levels with ℓ=0 correspond to vibrational band origins.

$v_{1}$	$v_{2}$	ℓ	$v_{3}$	*J*	p	*n*	*E* (cm ${}^{-1}$)
$\tilde{X}{\;}^{1}\Sigma_{g}^{+}$							
0	0	0	0	0	e	118	0.0
0	1	1	0	1	e	13	63.8533045(4)
0	2	0	0	0	e	4	132.795(6)
0	2	2	0	2	e	10	133.939(6)
0	3	1	0	1	e	2	207.873(6)
0	4	0	0	0	e	2	286.558(6)
0	4	2	0	2	e	4	288.156(6)
0	4	4	0	4	e	1	291.042(16)
0	5	3	0	3	e	3	373.463(5)
0	5	5	0	5	e	2	377.523(5)
1	0	0	0	0	e	4	1224.52(1)
0	1	1	1	1	f	3	2078.957(3)
0	2	2	1	2	f	2	2128.302(8)
0	4	4	1	4	f	2	2251.089(15)
0	5	5	1	5	f	2	2322.741(7)
0	5	3	1	3	f	1	2329.286(9)
2	0	0	0	0	e	1	2436.1(6)
1	1	1	1	1	f	2	3330.9496(5)
0	2	2	2	2	e	1	4081.9(5)
0	4	2	2	2	e	1	4199.9(5)
0	6	2	2	2	e	1	4333.9(5)
0	8	2	2	2	e	1	4486.7(5)
1	0	0	2	0	e	2	5268.399(2)
2	0	0	2	0	e	2	6460.663(2)
3	0	0	2	0	e	2	7635.526(18)
4	0	0	2	0	e	1	8799.5(6)
$\tilde{A}{\;}^{1}\Pi_{u}$							
0	0	1	0	1	e	13	24675.882(2)
0	0	1	0	1	e	9	24676.744(5)
0	0	1	0	1	e	1	24678.68(2)
0	1	2	0	2	e	9	24937.126(2)
0	0	1	1	1	f	1	25217.617(8)
0	1	2	1	2	f	3	25470.72(1)
0	2	2	1m	2	e	2	25567.995(8)
0	1	1	1	1	e	2	25695.21(8)
1	0	1	0	1	e	10	25762.266(2)
0	2	2	1p	2	e	1	26062.290(8)
1	2	1	0	1	e	2	26128.065(2)
1	0	1	1	1	f	1	26290.438(8)
0	0	1	2	1	e	4	26347.026(2)
0	3	1	1	1	e	2	26438.48(16)
1	1	1	1	1	e	2	26770.29(4)
2	0	1	0	1	e	2	26845.82(10)
0	5	1	1	1	e	2	27166.24(15)
0	3	3	3	3	e	2	28599.89(8)
1	1	1	3	1	e	2	28957.10(4)
0	5	1	3	1	e	2	29294.16(8)
1	3	1	3	1	e	2	29660.30(4)

### 4.3. Vibrational band origins (VBO)

There seems to be only rather limited compilations of accurate experimental vibrational band origins, see [35, 77]. Conversely Rohfling [37] gives vibrational band origins for many states obtained from dispersed fluorescence spectra but these band origins typically have an uncertainty of a few cm ${}^{-1}$, which means that they are probably less reliable than the results of the high accuracy *ab initio* calculations of Schroeder and Sebald [34, 77].

The MARVEL procedure only provides information on physical energy levels which means that for ${\text{C}}_{3}$ vibrational band origins, defined as the energy a vibrational states with *J* = 0, are only available for states with ℓ=0. In general the lowest energy for each vibrational state is given by the level with J=ℓ. Table 3 gives a compilation of such levels obtained in the current work. Notably those states with determined by only a single transition have higher uncertainties than the other levels.

Tables 2 and 3 are notable for the lack of data on the overtone states of the $v_{1}$ symmetric stretch. Direct transitions to these states from the vibrational ground state are forbidden and they largely remain poorly determined. Thus, for example, neither of the previous compilations 18ScDoSeZh [34] nor 23MaQiDoPi [35] give an empirical value for the symmetric stretch, $v_{1}$, band origin.

## 5. Summary and conclusions

A comprehensive analysis of the available spectroscopic data for the $\tilde{X}{\;}^{1}\Sigma_{g}^{+}$ and $\tilde{A}{\;}^{1}\Pi_{u}$ states of ${\text{C}}_{3}$ is presented which yields a list of 1887 empirical energy levels and associated uncertainties. The energy levels form an important starting point for generating a comprehensive line list for ${\text{C}}_{3}$ which is planned within the framework of the ExoMol project [78]. Such a line list is urgently needed for a variety of applications not least of which is the study of carbon-rich cool stars [79]. The first step in the generation of this line list will be the use of the empirical energy derived here as input to the fit of semi-empirical potential energy surface.

Although I was largely successful in recovering the transition wavenumbers from standard high resolution spectroscopic studies, there is another set of experiments for which it provided impossible to include most of the data. Two photon techniques such as laser induced fluorescence (LIF) or stimulated emission pumping (SEP) were extensively used two or so decades ago to study higher lying ro-vibrational levels in the $\tilde{X}{\;}^{1}\Sigma_{g}^{+}$ ground state [19, 21, 22, 37, 39, 40]. The very flat bending potential in ${\text{C}}_{3}$ makes this technique particularly appropriate for accessing highly excited bending states in this molecule. Unfortunately nearly all of the transition wavenumbers recorded in these studies, which in most cases were not particularly high resolution, are no longer available. Performing similar experiments with modern, high resolution techniques, would provide a very valuable source of data on states which are currently not well characterised but will be thermally occupied in hot environments such those found in the atmospheres of cool stars.

## Data Availability

The data compiled and generated in this article is given in the supporting information in the form of a MARVEL input transitions file, a MARVEL output energy file and the segment file necessary to run the MARVEL input under MARVEL4; this file gives the units for the transitions by data source.
